# Lehren aus der Russischen Grippe für das Endspiel der derzeitigen Pandemie – die Exitstrategie für Deutschland: Eine Streitschrift

**DOI:** 10.1007/s11553-021-00882-5

**Published:** 2021-08-05

**Authors:** Josef Weigl

**Affiliations:** MTropPaed, DTMH, DipEPP, Gesundheitsamt Plön, Schleswig-Holstein, Hamburgerstr. 17, 24306 Plön, Deutschland

**Keywords:** Atemwegsviren, Endemisch, Erstinfektion, Mutanten, Zweitinfektion, Airway viruses, Endemic, Mutants, Priming infection, Secondary infection

## Abstract

**Hintergrund:**

Seit dem Beginn der SARS-CoV-2-Pandemie wurde in Deutschland noch nie eine konkrete Strategie formuliert. Einzelne Themen verselbstständigen sich immerfort und die Begründungen für den Lockdown, nämlich die Reduktion der Mortalität bei den vulnerablen Personen- und Altersgruppen und die Gefahr einer erhöhten Letalität bei Überlastung des Gesundheitswesens, allen voran der Intensivstationen, geraten aus den Fokus.

**Methode:**

Auf der Basis von Erkenntnissen, die bereits vor dieser Pandemie vorlagen und solchen, die bisher akkumuliert wurden, wird eine Refokussierung vorgenommen und eine Exitstrategie für Deutschland entwickelt.

**Ergebnisse:**

Das eigentliche Ziel in der Pandemie ist, die Erstinfektion der Bevölkerung mittels Wildvirusinfektion oder Impfung möglichst schnell zu überwinden und die Herdenimmunitätsschwelle zu erreichen. Nur so kann dem Virus sein Gefahrenpotenzial genommen und eine neujustierte endemische Lage erreicht werden. Die Russische Grippepandemie 1889–1892 durch CoV-OC43, heute eine pandemische Narbe von damals, ist dazu das Modell. Die aktuell erfolgreiche Impfstoffentwicklung in nie dagewesener Geschwindigkeit ist eine historische Gnade.

**Schlussfolgerungen:**

Die Russische Grippepandemie war die zuletzt größte Coronaviruspandemie. Nach einem Impfangebot an die vulnerablen Gruppen („Targetpopulation“) ist der Lockdown schnellst möglich aufzuheben, das Impfangebot für Erwachsene ohne etablierte Risikofaktoren fortzusetzen und parallel dazu der Rest der Bevölkerung schnellst möglich durchseuchen zu lassen. Trotz erfolgreicher Impfstoffentwicklung drängt die Zeit, um weiteren Verwerfungen vorzubeugen.

## Abstract (en)

**Background:**

Since the beginning of the severe acute respiratory syndrome coronavirus type 2 (SARS-CoV-2) pandemic, Germany never formulated an explicit strategy so far. Topics continuously become disconnected from their origin and the justification for the lockdown gets out of focus, i.e. the reduction of mortality in the vulnerable population and age-groups, and the risk of an increased case fatality when the healthcare system—primarily intensive care capacity—would become overwhelmed.

**Methods:**

On the basis of knowledge which was already available before the current pandemic and knowledge which has been accumulated so far, refocusing and developing of an exit strategy for Germany is attempted.

**Results:**

The central goal in pandemics is to overcome the primary infection of the population by wild type infection or vaccination as quickly as possible and to achieve the herd immunity threshold. Only in this way can the virus lose its dangerous potential and a new endemic equilibrium can be achieved. The Russian Flu Pandemic from 1889–1892 by CoV OC43, today a pandemic scar from that time, can function as a role model. The successful vaccine development in unprecedented speed is a grace of historic dimension.

**Conclusions:**

The Russian Flu Pandemic was the most recent and largest coronavirus pandemic. After offering vaccination to all vulnerable groups (target population), the lockdown has to be ended as soon as possible. Vaccinations should continue to be offered to adults without established risk factors and the remaining population should be primed by infection with the wild virus. In spite of the rapid, successful development of a vaccine, time to avoid further disruptions is running out.

## Einleitung

Seit dem Beginn der SARS-CoV-2-Pandemie wurde in Deutschland noch in keiner Phase eine konkrete Strategie formuliert. Aus den Entscheidungen auf Bundesebene konnte man nur folgern, was man jeweils nicht wollte. Ansonsten war das Vorgehen bisher bestenfalls ein Vorgehen auf Sicht, mit möglichst geringem Risiko und der möglichst niedrigsten Mortalität. Sehr früh hatte man sich gegen das Konzept der Herdenimmunität durch die Wildvirusinfektion entschieden, denn das Risiko und die Systembeanspruchung erschienen zu hoch, und man setzte damit alles auf eine Karte einer erfolgreichen Impfstoffentwicklung. Nun ist den bisherigen Entscheidungen wider Erwarten ein historischer Glücksfall beschieden worden. In nie da gewesener Geschwindigkeit konnte die Impfstoffentwicklung erfolgreich vorangetrieben werden, und die Impfstoffentwicklung scheint nach bisherigen Erkenntnissen mit einer Wirksamkeit je nach Studienendpunkt von bis zu 95 % weit über den Erwartungen (Wirksamkeit von 30 % als unteres Limit eines 95 %-Konfidenzintervalls) auch geglückt zu sein. Seit Ende Dezember 2020 wird auch in Deutschland gegen SARS-CoV‑2 geimpft. Für eine abschließende Beurteilung ist es allerdings derzeit noch zu früh. Schwer auszumalen wäre wohl gewesen, wie die Lage derzeit wäre, wenn noch keine Impfstoffe zur Verfügung stünden und Deutschland in die Sackgasse zwischen Lockdown, wirtschaftlichen Kollaps und Aufkündigung des Vertrauens der Bevölkerung geraten wäre [22].

Mit der hier vorgelegten Arbeit soll aufgezeigt werden, wie, ausgehend von dem natürlichen Verlauf von Coronaviruspandemien und dem wissenschaftlichen Kenntnisstand zu Atemwegsviren, auf einige Grundprinzipien geschlossen werden kann. Diese wiederum sind dann essentiell, um realistische Planungen bis hin zu einer Strategie, ja Exitstrategie, für Deutschland zu entwickeln.

## Methoden

Auf der Basis von wissenschaftlichen Erkenntnissen, die bereits aus der Zeit vor der jetzigen Pandemie stammen, soll eine „Physiologie“ von Coronaviruspandemien beschrieben werden und in Zusammenschau mit den Erkenntnissen zur Epidemiologie, Virologie, Infektiologie und Vakzinologie von Atemwegsviren beim Menschen i. Allg. eine Folgenabschätzung vorgenommen werden. Aus diesen Gesichtspunkten ergeben sich dann offensichtliche Entscheidungsgrundlagen oder Kernvariablen für eine definitive Strategie für Deutschland. Es gilt, noch weitere Kollateralschäden für unser Land zu vermeiden. Für die Strategieentwicklung werden zudem die bisherigen Beobachtungen zu Public-Health-Maßnahmen, insbesondere die sog. nicht-pharmazeutischen Interventionen (NPI), aber insbesondere auch die seit Dezember 2020 möglichen Impfungen, einbezogen.

Zentral bei der Strategieentwicklung ist die Erkenntnissicherheit. Diese wird von den sog. „confoundern“, den „eigentlichen Verursachern“ eines Effekts, der durch andere Einflussgrößen (Variablen) vorgetäuscht wird, unterlaufen. Die Maßnahmen innerhalb einer Strategie sind auf ihre Auswirkungen auf die Zeitachse, die Nebenwirkungen und ihre Effekte flussabwärts bis hin zu einem „contingency plan“ zu betrachten. Die Auswirkungen der Strategie auf die Gesundheit der Bevölkerung und auf die Wirtschaft und Finanzen sind schließlich Kosten-Nutzen-Betrachtungen zu unterziehen. An einigen Stellen erlaube ich mir politisch zu werden, denn die Politik mischt sich ja auch in die Fachebene ein.

## Ergebnisse

### Russische Grippepandemie 1889–1892

Bisher galt die sogenannte Russische Grippe aufgrund der klinischen Symptomatik als von einem Influenzavirus verursacht. Ein H2- bzw. ein H3N8-Virus wurde bisher favorisiert. Man vermutete dahinter bereits ein Dogma eines Abwechselns der drei beim Menschen bekannten Hämagglutinine (H1, H2, H3) in immer gleicher Reihenfolge [13].

Über die etablierte Technik der molekularen Uhr konnte mittels Mutationsfrequenz bei Isolaten aus eingelagerten Proben aus der Arbeitsgruppe von van Ranst aus Löwen nachgewiesen werden, dass das CoV-OC43 aus dem bovinen CoV (BoCoV) im 19. Jahrhundert hervorgegangen ist [18]. Das BoCoV stammt vermutlich aus der Maus und das Rind diente als letzte Zwischenstufe auf dem Weg zur Adaptation an den Menschen [4]. Nach Deletion und Mutation schaffte das BoCoV als CoV-OC43 den Speziessprung vom Rind auf den Menschen und löste 1889 die sog. Russische Grippepandemie aus [18]. Das heißt, die Russische Grippe war in echt keine Grippepandemie, sondern eine Coronaviruspandemie. Nach bisherigen Erkenntnissen nahm diese Pandemie ihren Ausgang in der Region des heutigen Buchara in Usbekistan, damals unter Verwaltung des zaristischen Russland und deshalb als „Russische Grippe“ bezeichnet [13]. Von dort aus machte sie sich auf und erreichte nach ca. 7 Monaten Europa. Sie gilt als die erste, im Detail beschriebene Pandemie mit dem klassischen Verlauf in drei Wellen, mit der zweiten Welle als der stärksten. Es wird angenommen, dass weltweit 300.000 bis 1 Mio. Menschen dieser Pandemie zum Opfer fielen. Die detailliertesten epidemiologischen Daten gibt es aus Massachusetts [5]. Bei einer typischen U‑förmigen Altersverteilung stieg die Mortalität mit dem Alter und übertraf bei über 55-Jährigen die altersstratifizierte Mortalität der Spanischen Grippe bei weitem. Bei 80-Jährigen betrug die Mortalität ca. 1500 pro 100.000. Die absoluten Todeszahlen waren entsprechend der damaligen Bevölkerungsstruktur aber geringer als zum Zeitpunkt der Spanischen Grippe oder gar heute. Darüber hinaus gab es damals noch keine Intensivmedizin, Antibiotika oder Impfstoffe. Die Bezeichnung „Russische Grippe“, ähnlich der Bezeichnung der Spanischen Grippe (1918–1920) oder der Mexikanischen Grippe (2009/2010), ist somit irreführend und dies hier noch mehr, denn der ursächliche Erreger war kein Influenzavirus, sondern ein Coronavirus.

### Endemisches CoV-OC43 als pandemische Narbe von 1889–1892

Nachdem die Russische Grippepandemie über die Erde hinweggegangen war, wurde CoV-OC43 zu einem endemischen Atemwegsvirus beim Menschen und infizierte von da aus die verbliebenen, empfänglichen Erwachsenen und v. a. neu in die humane Population hineingeborene Kinder. Wie auch bei den anderen Atemwegsviren in der endemischen Phase dominieren seitdem Erkrankungen bei Kindern und evtl. fragilen Älteren mit Grundkrankheiten, die generell für das Bewältigen von Atemwegsinfektionen ein Risikofaktor sind [20, 27]. Für die meisten Erwachsenen blieben sie bisher vom Gesundheitssystem unbemerkt, auch mangels diagnostischer Anstrengungen und mangels Bewusstsein. Im infektionsepidemiologischen Forschungsnetzwerk (PID-ARI.net 1999–2005) wurde von 2002–2005 im Rahmen der erregerspezifischen Surveillance auch auf CoV-OC43 in der bis zu 19-valenten m‑RT-PCR untersucht [25]. Bei 3000–3500 Proben pro Jahr bei Kindern und Jugendlichen aus den Standorten Kiel, Mainz und Freiburg konnte bei 2,7–3,9 % CoV-OC43 bzw. 229E nachgewiesen werden. Bei vorwiegend hospitalisierten Kindern und wenigen ambulanten Fällen aus assoziierten pädiatrischen Praxen konnte ein Altersmedian von 1,5 Jahren für hospitalisierte Kinder und von 3 Jahren für ambulante Fälle errechnet werden. Der Anteil von Koinfektionen mit anderen Erregern lag bei den beiden Coronaviren über 50 %. Dies verweist auf eine geringere Rolle der Coronaviren als kausales Pathogen und auf ein noch längeres Ambulieren bis zur Erkrankung. Diese Viren wurden jährlich ohne einen besonderen Rhythmus nachgewiesen und die Altersverteilung war wie bei anderen Atemwegsviren auch. Bei Erwachsenen gelten Coronaviren meist als sog. Erkältungsviren mit meist weniger gefährlichen Verläufen als bei Kindern. Infektionen bei Kindern der genannten Altersgruppen unterscheiden sich von denen im Erwachsenenalter dadurch, dass die Infektionen bei Kindern überwiegend sog. Primärinfektionen sind, die generell einen gefährlicheren Verlauf haben können als die bei Erwachsenen, die Zweit- oder Folgeinfektionen sind. Gefährlicherer Verlauf bedeutet hier das Risiko für eine Krankenhausaufnahme. Das junge Alter und die immunologische Naivität spielen hierbei eine Rolle. Das heißt, für Kinder ist immer Pandemie, auch in Nicht-Pandemiezeiten, denn sie erfahren ja die Erstinfektionen bei Atemwegsviren. Die Erwachsenen sind durch die bereits durchgemachte Erstinfektion oder sogar mehrerer Infektionen im Lebenslauf gewappnet, auch gegen die, bei endemischen Erregern ebenso fortlaufend entstehenden Mutanten aufgrund der Kreuzimmunität durch die Vorinfektionen mit dem entsprechenden Erreger und der stattgehabten Immunreaktion.

### Metapher des Überquerens des Flusses namens Erstinfektion

In einer Pandemie, auch in der jetzigen, ist das Grundproblem, dass auch die Erwachsenen immunologisch naiv für den neuen Erreger sind und durch die Erstinfektion (immunologisch: „priming“) hindurchkommen müssen, nicht nur die Kinder. Mit zunehmendem Alter kann aber die immunologische Prägung ein Risikofaktor werden, insbesondere wenn sie nicht zur Immunität bzw. Kreuzimmunität wie bei den endemischen Coronaviren führt, sondern eine immunologische Unter- (z. B. insuffiziente Interferonantwort) oder Über- („cytokine storm“) oder Fehlreaktion („antigenic sin“) auslöst. Durch die Erstinfektion zu kommen, kann mit dem Überqueren eines Flusses verglichen werden. Der Fluss, durch den man muss, ist die Erstinfektion. Der schwere Verlauf wäre das Abdriften nach flussabwärts. Der Weg zum gegenüberliegendem Ufer geht durch die Erstinfektion, entweder in Form der Wildvirusinfektion mit einer sehr breiten Immunantwort oder einer artifiziellen Immunantwort, ausgelöst durch eine Impfung, die schmäler ausfällt, je nach Impfstoffkonstrukt. Im Falle von SARS-CoV‑2 beschränken sich die bisher zugelassenen Impfstoffe alleinig auf das Spike-Protein oder die „receptor binding domain“ des Spike-Proteins als Impfantigen. Entsprechend dem oben Beschriebenen basiert das Impfkonzept darauf, dass man erwartet, dass durch die Impfung mit dem Spike-Protein eines gegebenen Isolats eine Kreuzimmunität gegen aufkommende Mutanten bzw. Varianten erzielt wird, wie es in der breiteren Immunantwort bei der Wildtypinfektion ohnehin meistens gegeben ist. Das ist der springende Punkt.

### Impfen (Durchimpfungsrate) und Wildvirusinfektion (Durchseuchung)

Bei der Wildvirusinfektion im pandemischen Geschehen wie der Russischen Grippe wird die Bevölkerung in den drei genannten Wellen weitgehend (an die Herdenimmunitätsschwelle –„herd immunity threshold“ heranreichend) durchseucht und ist nach überstandener Erstinfektion ausreichend immun gegen aufkommende Mutationen des nunmehr endemischen Erregers. Der Erreger muss aber mutieren, wenn er in der menschlichen Population nach abgelaufener Pandemie und Durchseuchung der vormals naiven Population weiter existieren will, denn er braucht empfängliche Menschen für seine Replikation und Weiterverbreitung. Wie oben beschrieben, erreicht der Erreger mit Mutationen Zweit- oder Folgeinfektionen, und dies zu besonders günstigen Konditionen für den Wirt und sich selbst, denn das Virus wird durch die weniger schwer Kranken (durch die Kreuzimmunität) besser verbreitet. Als Beiwerk werden dadurch dann auch die wenigen neuen, naiven Opfer (sprich Kinder), die in die Populationen nach der Pandemie oder später fortlaufend geboren werden, gefunden. Der Preis für die menschliche Population ist dann viel geringer als in der pandemischen Situation. Zudem kommt der große Vorteil, dass Kinder meist, wie auch in dieser Pandemie, bei einem neuen Erreger deutlich besser abschneiden als Erwachsene.

### Die Strategie für Deutschland hin zu einer neujustierten endemischen Lage

Nun gilt es, die wissenschaftlichen Eckpfeiler rund um Atemwegsviren i. Allg. und der Coronaviren im Besonderen in eine Strategie zu gießen, die nachhaltig tragen kann. Nachdem der Herdenimmunitätsansatz durch die Wildvirusinfektion nicht genommen wurde und glücklicherweise die Impfstoffentwicklung so zeitnah geglückt scheint, gilt es, diese nunmehr zu kapitalisieren und die Bevölkerung unter diesen, vom Schicksal gut gemeinten, Bedingungen auf das andere Flussufer zu bringen. Auf dem Weg dorthin sollten aber die vorgenannten Grundtatsachen nicht übergangen werden, sondern immer wieder rekapituliert werden. Wichtig dabei ist, dass von einer Impfung zwar eine Reduktion der Übertragung zu erwarten ist, da bei einem Geimpften, wie bei einer Zweitinfektion auch, die Viruslast nicht so hoch steigt und von viel kürzerer Dauer ist als bei der Erstinfektion, aber dennoch Übertragungen stattfinden können. Das wirksame Zeitfenster einer Übertragung des Wildvirus bei Geimpften ist als geringer zu erwarten – z. B. 3–5 Tage anstatt 10–14 Tage nach Erstinfektion. Eine Infektion mit dem Wildvirus löst beim Geimpften eine Booster-Reaktion aus – soweit das angedeutete Grundprinzip der Epidemiologie von Atemwegsviren. Aufgrund des Geschilderten und der Tatsache, dass dieser Erreger maximal eine Teilimmunität verleiht, ist es aber unrealistisch zu erwarten, dass die derzeitigen Impfstoffe eine sog. sterile Immunität induzieren können, wie dies z. B. bei Masern bzw. nach Masernimpfung (MMR) der Fall ist. Ganz im Gegenteil, da die sog. Erstgenerationsimpfstoffe auf einer sehr schmalen Antigenbasis beruhen, ist ein „immune-escape“ durch Mutanten möglich. Allerdings besteht auch hier die begründete Hoffnung einer ausreichenden Kreuzprotektion sowohl durch die Breite der humoralen, der B‑Zell-, als auch der T‑Zell-Immunität und des immunologischen Gedächtnisses, auch wenn die Basis der Immunantwort nur das Spike-Protein ist. Wichtig wäre es jetzt, ganz schnell die sog. vulnerablen Personen (d. h. Personen- und Altersgruppen mit erhöhter Mortalität = „Targetpopulation“) zu impfen, um dann, unter Fortsetzung der Impfkampagne, den Rest der Bevölkerung schnell auch auf natürlichem Weg durchseuchen zu lassen. Dies würde dem Erreger insgesamt seine Gefährlichkeit nehmen, denn die Anflutungsgeschwindigkeit und die Größe der Welle an Erstinfizierten ist das gefährliche Moment in einer Pandemie, auch der derzeitigen. Je schneller der Anteil empfänglicher Personen reduziert wird, umso schneller verlieren die Pandemie und das Virus seine Potenz. Der Stolz auf die verhandelten Impfstoffpreise von nunmehr einem Gegenwert eines Antigenschnelltests ist da völlig unverständlich, von den indirekten Kosten eines Lockdowns für die Gesellschaft gar nicht zu sprechen. Die jetzt losgetretene Teststrategie für alle Bürger verbrennt Geld, macht das Management via NPI möglicherweise weniger effizient, und das Geld wäre in einen Impfstoff besser investiert gewesen. Um in Anbetracht der erheblichen Entwicklungsverzögerung des mRNA-Impfstoff der Firma CureVac (Tübingen) dennoch den schnellen Zugang zu diesem Impfstoff zu ermöglichen, sollte eine Notfallzulassung mittels „bridging“ zu den Wirksamkeitsdaten der beiden bereits etablierten mRNA-Impfstoffen von BioNTech (Mainz) und Moderna (Boston) über Daten zur Immunogenität erwogen werden. Die unmittelbare Nutzung des Impfstoffs von CureVac sollte dann in Deutschland erfolgen, um nicht wieder zu erleben, dass Produkte, die mit deutschen Forschungsgeldern und Forschungsinitiativen entstanden sind, vornehmlich anderen zugutekommen. Dies allerdings aber nur, wenn CureVac mit einer signifikanten Impfstoffmenge aufwarten kann. Um der Bevölkerung endlich eine Perspektive zu geben, auf die sie sich einstellen kann, sollte man sich evidenzbasiert, wie bereits bei der Priorisierung durch die STIKO [20], auf die epidemiologischen Daten berufen und die beiden zentralen Ziele – Reduktion der Mortalität bei Vulnerablen und keine erhöhte Letalität durch Überlastung der Kliniken – im Fokus behalten und das Thema nicht in Eigendynamik erweitern auf einen Impfzwang, ein Impfzertifikat oder eine Routine- oder Zwangsimpfung bei Kindern und Jugendlichen. Die sog. „Targetpopulation“ wären die fünf bzw. drei Priorisierungsgruppen laut STIKO und Bundesministerium für Gesundheit [2, 20]. Dass Kinder unter 11 bzw. 14 Jahren nicht zum pandemischen Geschehen, sprich Übertragung auf andere, beitragen, sollte inzwischen Allgemeingut sein. Bei den Mutanten („variants of concern“ [VOC]), inklusive der britischen B 1.1.7 wird derzeit zwar behauptet, dass es anders wäre, aber hier sind „confounder“ naheliegend, denn das sich dahinter verbergende biologische Prinzip hat sich mit dem Auftreten der VOC nicht verändert. Hohe Infektionsraten bei Kindern belegen noch keine Übertragung durch diese. Wenn viele Variablen ineinandergreifen und ein „deconfounding“ nicht mehr sicher möglich ist, dann sind Daten aus Interventionsstudien von höchstem Wert. Soeben berichtete Ron Dagan (persönliche Mitteilungen), dass man in Israel einen drastischen Rückgang der Infektionen bei Kindern beobachtet, seitdem die Erwachsenen geimpft worden sind. Dies bestätigt, dass die bisherigen Beobachtungen einer unmöglichen oder ineffizienten Übertragung ausgehend von Kindern, auch bei der Variante B 1.1.7, der Fall ist und damit andere Verlautbarungen „confoundern“ aufgesessen sind.

Das Phänomen, dass Kinder SARS-CoV‑2 nicht oder sehr ineffizient übertragen, ist zwar auf den ersten Blick eine Überraschung in Vergleich zu anderen endemischen Atemwegsviren, aber dann auch wieder keine, wenn man bedenkt, dass in der endemischen Situation die Kinder von der Erstinfektion betroffen sind und diese zu einer längeren Virusausscheidung führt als eine Zweitinfektion. Somit ist die Erstinfektion hier der Confounder. Bei diesem Erreger spielen aber scheinbar die pandemische Situation als auch evtl. besondere pädiatrische Faktoren eine Rolle [3, 17]. Letztere sind wissenschaftlich noch nicht völlig schlüssig: die Antwort des natürlichen Immunsystems, allen voran die Interferonantwort, ist bei Kindern erheblich stärker als bei Erwachsenen [28], aber es könnten darüber hinaus noch ganz andere Mechanismen eine Rolle spielen, wie z. B. ein „small molecule“, das vor der Pubertät vorhanden bzw. aktiv ist (Chebrolu A., CNN 16.10.2020). Dieses „small molecule“ könnte die Tertiärstruktur des Spike-Proteins derart verändern, dass es nicht mehr an den ACE2-Rezeptor andocken kann. Die Kinder jetzt auch noch durch einen Impfzwang zu schleusen, wäre das Fatalste überhaupt und würde nicht der wissenschaftlichen und epidemiologischen Grundlage entsprechen, sodass den Entscheidungsträgern und Fachkollegen dringend zu raten ist, von derartigen Maßnahmen Abstand zu nehmen.

Teilimmune asymptomatische oder oligosymptomatische Erwachsene mit einer Virusausscheidung, die zwar geringer ist als bei einer Erstinfektion, die aber dennoch sehr wirksam sein kann über ihr erhebliches Kontaktmuster, zumindest in einigen Berufen und sozialen Situationen, wurden als Treiber der Verbreitung bei Atemwegsviren, auch der Influenza, bisher viel zu wenig erforscht. Die saisonale Grippe hat durch die Erstinfektionen bei Kindern mit längerer Ansteckungsfähigkeit das Bewusstsein diesbezüglich doch auch in eine vornehmliche Richtung, sprich Überbetonung der Kinder, verzerrt, denn das Letztere kann zwar leichter untersucht werden, aber die Rolle der Erwachsenen ist dabei unzureichend untersucht.

Nach dem Impfangebot an die vulnerable Bevölkerung auf freiwilliger Basis, an weitere, die in den Priorisierungsstufen genannt sind und ggf. auch an weitere Personen, die sich für vulnerabel halten oder Befürchtungen wegen des Long-COVID-Syndroms haben, sollten die Beschränkungen bzw. die Lockdowns so schnell wie möglich aufgehoben werden und die Durchseuchung (Immunisierung) der Restbevölkerung nicht weiter verzögert werden. Die Auswirkungen auf die Bevölkerung, allen voran der Kinder, die Wirtschaft und Finanzen sind jetzt schon unabsehbar. Die immensen Geldsummen, die bisher verbrannt wurden, müssen spätestens nach der Bundestagswahl beglichen werden.

## Diskussion

Bisher wurde in dieser Pandemie auch von Medizinern oft so argumentiert, als ob bei SARS-CoV‑2 alles neu wäre und keine oder weitestgehend keine validen Vorerkenntnisse der Wissenschaft vorlägen, die Entscheidungen und Handlungen leiten könnten. Dass die derzeitige Coronaviruspandemie nicht die erste in der Menschheitsgeschichte sein kann, ist alleine schon aus dem Grunde anzunehmen, dass bereits mindestens vier sog. endemische Coronaviren beim Menschen vorkommen. Die beiden zuerst bekannten Coronaviren beim Menschen waren 229 E und OC43, die 1966 [8] bzw. 1967 [11] erstbeschrieben wurden. In 2003 und 2004 folgten dann NL63 bzw. HKU1, wobei NL63 einen gemeinsamen Vorfahren mit 229E hat [4, 14]. Wie am Beispiel von CoV-OC43 gezeigt wurde, haben vermutlich alle eine Pandemie, oder zumindest weite Epidemien, nach sich gezogen. Selbst vor 1889 gab es schon zahlreiche Pandemien bis weit vor unsere Zeitrechnung wie die Seuche von Thukydides (430 v. Chr); [9]. und alte chinesische Aufzeichnungen zeigen [7]. Coronaviren können bis 400 v. Chr. datiert werden [12]. CoV-NL63 wird auf das 10. bis 11. Jahrhundert und CoV-229E auf das 17. bis 18. Jahrhundert datiert [12, 14]. Die Technik der molekularen Uhr gilt heutzutage in Abhängigkeit der Probenanzahl über die Zeit als relativ präzise, ist gerade für die Paläovirologie von RNA-Viren von höchstem Wert und konnte u. a. auch belegen, dass Masern ca. zur Zeitenwende auf den Menschen übergegangen sind [16]. Die Antoninische Pest (165–180 n. Chr.), die bisher den Pocken zugeordnet wird, könnte sehr wohl durch Masern verursacht gewesen sein, denn früher wurde bei exanthemischen Krankheiten bis ins 8. Jahrhundert nach Christus nicht zwischen Masern und Pocken unterschieden [15]. Die Russische Grippe zeigt, dass „Grippe“ eben nicht automatisch verursacht durch ein Influenzavirus bedeutet, sondern dass damit zuerst einmal nur eine respiratorische Infektion gemeint ist. Die Russische Grippe belegt erstmals in der Medizingeschichte das Grundmodell einer Pandemie in drei Wellen. Allerdings wurde keine Pandemie bisher so derartig durch die Menschheit beeinflusst wie die jetzige, im positiven und im negativen Sinne. Ganz gewiss war auch keine bisher so teuer. Jetzt beginnt das Problem bereits bei der Begriffsfassung, denn momentan befinden wir uns in der 2. Welle, die in ihrer Intensität je nach Härte des Lockdowns moduliert wird. Fälschlicherweise wird das derzeitige Aufflackern nach den Lockerungen als 3. Welle bezeichnet. Von einer erneuten Welle spricht man aber nur, wenn es über längere Zeit nahezu keine Fälle gegeben hat, die Pandemie quasi eine Pause einlegte. SARS-CoV‑1 wurde zwar 2003 in viele Länder getragen, aber es kam zu keinem pandemischen Geschehen in Wellen, denn der Erreger hatte außerordentlich günstige Voraussetzungen für eine Kontrolle mittels NPI, da eine Ansteckung erst möglich war, nachdem der Patient schon erkrankt war, d. h. keine Präpatenzzeit vorgelegen hat [6, 23].

Das Krankheitsbild einer Grippe ist unspezifisch und die Krankheitsbilder der meisten viralen Atemwegsinfektionen können klinisch nicht treffsicher unterschieden werden. Durch die drei Influenzapandemien im 20. Jahrhundert dominierte die Influenzavirusforschung die Forschung zu Atemwegsviren und das Bewusstsein, sodass man erst durch die Untersuchungen von Vijgen et al. [18] auf die alternative Hypothese von CoV-OC43 in der Ätiologie der Russischen Grippe kam, an der für den Autor kein Zweifel mehr besteht. Es war ein Glücksfall, dass wenigstens seit den 1960er-Jahren Isolate über mehrere Jahre hinweg vorlagen, nach denen man die molekulare Uhr justieren konnte. Seit dem Auftauchen von SARS-CoV‑1 im Jahre 2003, gefolgt von MERS 2011, hat die Forschung an Coronaviren Fahrt aufgenommen und der seitdem umfangreiche Corpus an wissenschaftlicher Literatur hat sehr wohl die Potenz von Coronaviren und das Arsenal an Coronaviren in ONE Health dargelegt, die dem von Influenzaviren nicht nachsteht. Im Rahmen des Infektionsschutzes und der Seuchenkontrolle scheint jetzt der Hauptunterschied zwischen den beiden Virusgruppen v. a. noch in der potenziellen Anflutungsgeschwindigkeit gegeben zu sein, die bei Influenzaviren aufgrund der kürzeren Tg (Generationszeit bzw. Serienintervall) stärker ist als bei Coronaviren, trotz des höheren R_0_ bei letzteren. Die erheblich kürzere Tg bei Influenzaviren ist hier zentral und könnte der Hauptmechanismus sein, über den VOC momentan in der noch weitgehend SARS-CoV-2-naiven Bevölkerung gefährlich werden könnten. Die Erkenntnisse aus grundlegenden Forschungen zur Epidemiologie und Virologie sind von hohem Wert und die seinerzeit nicht weitere Förderung des Forschungsnetzwerkes PID-ARI.net scheint aus heutiger Sicht nochmals unverzeihlicher [24].

Bei der Beurteilung der Schwere und der Komplikationen von Primärinfektionen im Vergleich zu Zweit- oder Folgeinfektionen muss unterschieden werden zwischen Immunologie und Anfälligkeit durch pathophysiologische Fragilität im Alter mit einer höheren Prävalenz an Grundkrankheiten (Alter und Grundkrankheiten haben eine hohe Koliniarität). Ob man immunologisch naiv ist oder bereits geprimt, macht einen großen Unterschied in der Viruslast und der Pathogenität. Hiervon muss die schwächere Physiologie bei Grundkrankheiten im Allgemeinen und im hohen Alter im Besonderen unterschieden werden. In der Pädiatrie ist es umgekehrt und die Alterskomponente wirkt in umgekehrter Richtung, umso mehr, je jünger man ist [25, 26]. Bei Erstinfektionen ist das Hospitalisierungsrisiko erhöht. Dass Kinder im Vergleich zu Erwachsenen bei Erstinfektionen mit Atemwegsviren deutlich besser abschneiden, liegt an der natürlichen Immunantwort (Interferone) bzw. keiner überschießenden oder unterreagierenden adaptiven Immunantwort, außer bei den wenigen Fällen an MIS‑C („multisystem inflammatory syndrome in children“) [28]. Bei SARS-CoV‑2 kommt noch die Rezeptorenausstattung als wissenschaftlicher Grund hinzu [3, 17]. Ob hier noch weitere Phänomene wie das bereits erwähnte „small molecule“, wie es die 14-jährige Texanerin Anika Chebrolu entdeckt hat (CNN, 16. Oktober 2020), eine Rolle spielen, muss momentan noch offen bleiben. Das Phänomen, dass Kinder bei neuen Erregern deutlich besser wegkommen als Erwachsene, war auch schon bei der Spanischen Grippe sehr eindrücklich wie die Altersverteilung der Überlebenden in Inuit-Dörfern zeigte. Oft war der Dorfälteste nach der Pandemie nur 15 Jahre alt (John Oxford persönliche Mitteilung)!

Die Breite der ersten Immunantwort („priming“) ist bei der Wildvirusinfektionen um ein vielfaches größer als bei der Impfung und richtet sich bei ersterer nicht nur gegen das Attachmentprotein, hier das Spike-Protein, sondern gegen alle immunogenen Virusbestandteile, insbesondere auch gegen interne Antigene oder die sog. Nicht-Strukturproteine [19]. Deshalb ist die Kreuzimmunität gegen Mutanten des gleichen Erregers oder sogar gegen verwandte Coronaviren aus der ß‑Gruppe nach durchgemachter Wildvirusinfektion ausgeprägter als bei einer Impfung. Die bisher bekannten Atemwegsviren (außer bei saisonalen Grippeviren) verlieren nach der Erstinfektion ihren Schrecken und ihre Potenz für schwere Verläufe. Bei SARS-CoV‑2 ist das Gleiche zu erwarten [10]. Umgekehrt ist es aus Sicht des Erregers von großem Vorteil, soweit ein „escape“ zu schaffen, dass zwar die Übertragung möglich ist, aber die Erkrankungsschwere beim Wirt gering bleibt, sodass die Verbreitung durch einen infizierten Wirt voranschreiten kann und durch überwiegend leichte Verlaufsformen ohne ein Darniederliegen eine Verbreitung nicht unterbunden wird. Die Infektion mit dem Virus selbst ist für die Zukunft und aus der Perspektive der Population die bessere Versicherung als eine Impfung.

Die nach den begonnenen Impfungen gegen SARS-CoV‑2 schmälere Immunantwort ist bereits dadurch sichtbar, dass eine bereits stattgehabte Infektion mittels Spike-Protein-Vakzine nicht verhindert werden kann (Postexpositionsprophylaxe unmöglich!) und die Impfung, selbst wenn sie bereits einige Tage vorher stattgefunden hat, versagt, da keine internen Virusproteinantigene im Impfstoff enthalten sind [21].

### Kernpunkte für eine Strategie im Endspiel um die Coronapandemie 2020–2022

Die Pandemie zeichnet sich bisher u. a. dadurch aus, dass sich Themen in der Politik immer wieder völlig verselbstständigen und der Fokus auf den Ursprung eines Themas verloren geht. Nochmals, die Begründung für die Lockdowns war:

1. Die Mortalität bei den vulnerablen Gruppen und
2. Die erhöhte Letalität bei COVID-Infektionen bei einer Überlastung des stationären Versorgungssystems, allen voran der intensivmedizinischen Kapazitäten.

Darüber hinaus ist für das Bewusstsein wichtig, dass wir bei der Coronavirusbekämpfung über eine Hinhaltetaktik mittels NPI und Lockdowns sprechen bis genau zu dem Zeitpunkt, bis diese beiden Punkte weggefallen sind, und eben nicht über eine Eradikationskampagne gegen das SARS-CoV-2-Virus. Ab Aufhebung des Lockdowns auf der genannten Basis ist es von nachgeordneter Bedeutung, wie viel an Übertragung durch die Impfung gedrosselt wird. Dass diese nur gedrosselt werden kann, aber eben nicht aufgehoben wird, wurde oben dargelegt und liegt in der Natur des Erregers und seiner Immunologie. Es gibt nur wenige Beispiele in der Vakzinologie, in der die Impfimmunität einer Immunität, die durch eine Infektion mit dem Erreger selbst induziert wurde, überlegen ist, wie dies bei den antibakteriellen Konjugaktvakzinen gegen H. influenzae, S. pneumoniae und N. meningitidis der Fall ist.

Wie kommen wir möglichst schnell zu einer Populationsimmunität, die einem Zustand nach der Primärinfektion mit Erreichen der Herdenimmunitätsschwelle entspricht? Der Anteil der Menschen, die empfänglich für die Erstinfektion ist, muss schnellst möglich gesenkt werden, und das mit möglichst geringen Kollateralschäden auf allen Ebenen. Dann wird die Pandemie an Wucht und Potenz einbüßen und ab Oktober 2021 mit maximal 30–50 % an nicht explizit vulnerablen SARS-CoV-2-naiven Personen in eine 3. Welle zu gehen, sollte dann verkraftbar sein.

Was zu tun und zu lassen ist, ergibt sich aus der Tab. 1. Hat man den roten Faden, dann kann man auf nicht zielführende Nebenschauplätze wie ein Impfzertifikat verzichten. Den einzigen Sinn, den man nach dem Dargelegtem dafür noch sehen könnte, wäre, dass ein Zertifikatinhaber, der entweder geimpft ist oder eine Wildvirusinfektion durchgemacht hat, einem Gastgeberland signalisieren kann, dass er dem dortigen Gesundheitssystem wegen COVID nicht zur Last fallen könnte, da eine schwere Erkrankung dann unwahrscheinlich wäre. Aber alleinig dafür wäre das Instrument höchstens von einer sehr kurzen Nutzungsdauer.

**Tab. 1:** Eine Strategie für das Endspiel um die Pandemie 2020–2022 bei anhaltender Impfstoffknappheit

*Thema*	*Maßnahme*	*Beurteilung*
Impfstoffversorgung	Altersabhängig generell Einmaldosisschemen anwenden	Wirksamkeit bei unter 80-Jährigen (vorübergehend) ausreichend
	Keine Impfung nach gesicherter Erstinfektion	Rechtfertigung der Einmaldosis bei Genesenen eher durch die Zweifel, wie Sicher die Diagnose war als immunologisch
	Impfstoff von CureVac über „bridging“ mittels Daten zur Immunogenität schnell zulassen und in Deutschland prioritär verwenden	Mit Aufregungen ist zu rechnen, aber die Plausibilität liegt nahe, auch wenn der Ansatz für EMA und PEI unkonventionell ist
	Sputnik-V-Produktion in Deutschland oder Zukauf; die erste Komponente basierend auf Ad26 als Einmaldosisschema einsetzen	In Analogie zur Vakzine von Johnson & Johnson ebenso auf Ad26 und deren Monodosisschema. Das Studiendesign bestimmt ja über die Marktzulassung
	Impfzentren und Hausärzte einsetzen	Der Anspruch an die Handhabung des Impfstoffes muss berücksichtigt werden
Zweitinfektion bzw. Infektion nach Impfung	Vorrübergehende Beachtung bis Durch-impfungsraten in der „Targetpopulation“^a^ gesichert ist; z. B. via 5–7 Tage Isolierung. Nach Abschluss des Impfangebotes für Vulnerable, umgehend aus der Betrachtung ausklammern	Für Quarantänen ist die Beurteilungsbasis schwieriger, denn wie lange die Inkubationszeit nach Impfung oder bei der Folgeinfektion sein kann, ist unbekannt. Auch dieser Punkt wäre nach der Durchimpfung der „Targetpopulation“ obsolet
Kinder	Kinder 0 bis 11/14 Jahren völlig ausklammern, denn sie übertragen das Virus nicht effizient	Keine Impfempfehlung bei Kindern ohne diesbezüglichen bedeutende Grundkrankheiten
Jugendliche	Jugendliche 14 bis 18 Jahre und ihre Rolle als effiziente Überträger	Schnelle Durchseuchung auf natürlichem Wege; Inzidenz schwerer Verläufe gering

*EMA* European Medicines Agency (Amsterdam), *PEI* Paul-Ehrlich-Institut

^a^Die „Targetpopulation“ entspricht den bisherigen drei bzw. fünf Priorisierungsgruppen nach Impfverordnung bzw. STIKO [2, 20]

Public-Health-Maßnahmen müssen einfach (d. h. wenig komplex), robust und schlagkräftig sein. Die Misere der Impfstoffbeschaffung Deutschlands zusammen mit der EU soll hier nicht weiter vertieft werden, allerdings sollte man, nachdem die über 80-Jährigen weitgehend geimpft sind für jüngere Altersgruppen, wie im Vereinten Königreich auch, nun auf Einmaldosisschemen setzen und die zweite Dosis erst nachholen, wenn ausreichend Impfstoff vorhanden ist [1]. Bei der letzten Influenzapandemie 2009/2010 hielt man zu lange an dem Zweidosisschema von Pandemrix fest. In Pandemiezeiten sollte man starre Entscheidungswege vermeiden und das vorliegende, supportive Wissen in die Entscheidungen einbeziehen. Nachdem die Höchstbetagten bereits 2‑mal geimpft sind und für jüngere ausschließlich Einzeldosisschemen verwendet werden, könnte die „zweite Dosis“ ein Booster durch das Wildvirus oder die zweite Dosis eines Impfstoffs zu gegebener Zeit sein. Das immunologische Gedächtnis gilt und jede Dosis zählt, auch wenn die Impfabstände erhöht werden. Wie bereits erwähnt, sollte man jetzt dringend darüber nachdenken, die CureVac-Vakzine mittels „Bridging“-Daten zuzulassen, sollten sich die Studien noch weiter verzögern. Nachdem inzwischen umfängliche Erfahrung mit den mRNA-Produkten von BioNTech und Moderna akkumuliert wurden, wäre „bridging“ innerhalb der gleichen Plattformtechnologie die Begründung. Auch das Paul-Ehrlich-Institut müsste dazu flexibler werden, seine Ansätze überdenken und von rigiden Schemen Abstand nehmen, dass jeder Impfstoff für sich erst einmal die Wirksamkeit mittels einer Phase-III-Studie belegt haben muss, bevor „bridging“ über Daten zur Immunogenität zu einer Verkürzung des Zulassungsverfahrens oder der Zulassungsspezifitäten herangezogen werden können. An dieser Stelle wäre das kein „bridging“ innerhalb der gleichen Zulassung, sondern innerhalb der gleichen Plattformtechnologie. Für das Management künftiger Krisen ähnlich dieser Pandemie wäre dieser Schritt jetzt sinnvoll, auch aus erkenntnistheoretischen Gründen. Es wäre dann dafür zu plädieren, diesen Impfstoff vordringlich Deutschland zukommen zu lassen. Dem Autor ist bewusst, dass dies eine weitgehende (ja politische) Forderung ist, aber diese in Synopsis der Sachlage und der Kosten für einen noch längeren Lockdown bei schnell abfallender Resilienz und Vertrauen der Bevölkerung geboten ist. Deutschland hat hier nicht nur altruistische Aspekte bezüglich den anderen Mitgliedsstaaten der EU zu berücksichtigen, sondern auch die wirtschaftlichen Aspekte und die Tatsache, die sog. „Lokomotive“ der Wirtschaft der EU und der Hauptbeitragszahler für den Coronafond von 750 Mrd. € zu sein.

## Schlussfolgerungen

Die Russische Grippe war die bisher größte beschriebene Coronaviruspandemie. Die Erstinfektion ist bei einer naiven Gesamtbevölkerung das besonders Gefährliche. Diese zusammen mit dem medizinischen Versorgungssystem entscheidet über die Mortalität und Letalität. Die Anflutungsgeschwindigkeit und das Ausmaß einer weiteren Welle im Herbst 2021 würden bei einer hohen Durchimpfungsrate der vulnerablen Menschen und einer fortgeschrittenen Durchseuchung bis dahin erheblich abnehmen und die Einschränkungen oder Lockdowns könnten aufgehoben werden. Gegebenenfalls wäre, wenn überhaupt, maximal ein leichterer Lockdown nochmals notwendig, wenn die Durchseuchung der Restbevölkerung vor sich geht, ohne die das gegenüberliegende Ufer des Stroms der Erstinfektionen nicht erreicht werden kann. Für die Kinder ist immer Pandemie und wir können froh sein, dass sie bei SARS-CoV‑2 i. Allg. so gut wegkommen – sie sollten von weiteren Maßnahmen verschont bleiben und die Schulen in den Betrieb wie vor der Pandemie zurückkehren.

## Fazit für die Praxis

- Die zentrale Herausforderung in der Bewältigung der Pandemie ist, die Bevölkerung immunologisch schnellst möglich zu primen, durch eine Erstinfektion oder wenn möglich durch eine Impfung.
- Eine Kombination aus einer schnellstmöglichen Impfung der „Targetpopulation“ und der Durchseuchung der Restbevölkerung bei einem weiterhin möglichen Impfangebot soll dem Virus möglichst schnell sein Gefahrenpotenzial nehmen. Die Priorisierung ist beizubehalten.
- Nach dem Winter 2021/2022 könnte die neue endemische Lage erreicht werden. Ein pragmatisches und fokussiertes Handeln in einer konkretisierten Strategie ist überfällig.
